# Estenose do Enxerto de Veia Safena

**DOI:** 10.36660/abc.20200633

**Published:** 2020-09-18

**Authors:** Henrique Murad

**Affiliations:** 1 Universidade Federal do Rio de Janeiro Rio de Janeiro RJ Brasil Universidade Federal do Rio de Janeiro, Rio de Janeiro, RJ - Brasil; 2 Academia Nacional de Medicina Rio de Janeiro RJ Brasil Academia Nacional de Medicina, Rio de Janeiro, RJ – Brasil

**Keywords:** Oclusão do Enxerto Vascular, Trombose, Antiagregantes de Plaquetas, Aterosclerose//prevenção e controle, Fatores de Risco

A estenose ou oclusão do enxerto de veia safena (EVS) é o calcanhar de Aquiles para um uso mais liberal desse tipo de enxerto na cirurgia de revascularização miocárdica (CRM).

Durante o primeiro mês após a CRM, cerca de 10% dos EVS pode estar ocluída devido a falha da técnica cirúrgica ou trombose. No final do primeiro ano, 15% dos EVS podem estar ocluídos devido à hiperplasia intimal. Uma técnica cirúrgica perfeita, o manuseio cuidadoso da veia safena evitando a hiperdistensão do enxerto e o uso de aspirina por toda a vida (ou outro agente antiagregante plaquetário) são pontos importantes para a obtenção de uma veia patente.^1^

Após o primeiro ano, a etiologia mais importante para estenose ou oclusão do EVS é a aterosclerose, e após 10 anos apenas cerca de 60% dos enxertos de EVS são patentes. A estenose aterosclerótica do EVS é a indicação mais comum para repetir a CRM. Os fatores de risco para aterosclerose e estenose do EVS são semelhantes aos da artéria coronária nativa.^1^

Para prevenção da aterosclerose do EVS, utiliza-se a mesma estratégia usada para a aterosclerose da artéria coronária nativa, a saber, modificação de fatores de risco e medicação redutora de lipídios.

O artigo “Impacto dos índices aterogênicos na estenose do enxerto de veia safena”^2^ estudou 534 pacientes através de cineangiocoronariografia realizada pelo menos um ano após a CRM (mediana de 5,3 anos). Eles dividiram os pacientes em dois grupos: 1- EVS(+), com pelo menos um EVS com mais de 50% de estenose, com 259 pacientes; e 2-EVS (-) sem qualquer estenose do EVS, com 275 pacientes. Os autores estudaram os componentes lipídicos do plasma em ambos os grupos: colesterol total (CT), triglicérides (TG), lipoproteína de alta densidade-colesterol (HDL-C), lipoproteína de não-alta densidade-colesterol (não-HDL-C) e lipoproteína de baixa densidade-colesterol (LDL-C). A partir desses dados, eles calcularam o índice aterogênico plasmático (IAP) e o coeficiente aterogênico (CA). O IAP é calculado como o logaritmo da relação TG/HDL-C. O CA é calculado pela fórmula simples (CT-HDL-C)/HDL-C.

Nos resultados, eles mostraram que diabetes mellitus, hipertensão arterial, acidente vascular cerebral, insuficiência cardíaca e número de enxertos eram parâmetros clínicos independentes para estenose do EVS. Em relação aos parâmetros laboratoriais LDL-C, não -HDL-C, HDL-C, IAP e CA foram fatores independentes para estenose do EVS. Utilizando comparações pareadas de análise da curva ROC, eles não encontraram diferenças significativas entre IAP e CA, mas ambos foram melhores para prever estenose do EVS do que o HDL-C, LDL-C e não-HDL-C.

Tenho algumas observações e comentários sobre este artigo interessante, alguns deles já apresentados nas limitações do estudo:

1. Não houve *endpoint* clínico.
2. O uso de medicamentos hipolipemiantes foi baixo nos dois grupos, sendo 59,8% no grupo EVS(+) e 64,7% no EVS(-). Eles descobriram que o LDL-C médio dos pacientes estava acima do nível recomendado para prevenção secundária. Hata et al.,^3^ demonstraram que um tratamento agressivo com estatinas para atingir um nível de LDL<100 mg/dL diminui o número de enxertos afetados e a necessidade de repetir a revascularização .^3^
3. O número de enxertos foi um fator independente para estenose de EVS e o grupo EVS(+) teve mais enxertos implantados que o EVS(-). Como os autores consideram que o principal critério para ser incluído no grupo EVS(+) era ter pelo menos um enxerto com mais de 50% de estenose, questiono se estudar pacientes com o mesmo número de EVS não seria uma maneira mais precisa de comparação entre os grupos, já que se um número maior de enxertos for implantado, há maior probabilidade de pelo menos um deles ser estenótico.
4. Na técnica *no-touch* para a coleta de veias safenas, a veia é removida do leito com tecido circundante, sendo relatada patência de 90% em 8,5 anos;^4^ além disso, esse tipo de coleta de veias foi recomendado na Diretriz Europeia de 2018 para revascularização do miocárdio.^5^ Seria interessante conhecer o comportamento do IAP e CA nos resultados de médio prazo desses enxertos.
