# Supplementary material for: Estenose do Enxerto de Veia Safena
Source: Arq Bras Cardiol. 2020 Sep 18;115(3):545–6. [Article in Portuguese] doi: 10.36660/abc.20200633 (PMC9363084; doi:10.36660/abc.20200633)
Supplement: Translation

# Translation: Saphenous Vein Graft Stenosis

**Authors:** Henrique Murad

**Affiliations:** 1 Brazil Universidade Federal do Rio de Janeiro, Rio de Janeiro, RJ - Brazil; 2 Brazil Academia Nacional de Medicina, Rio de Janeiro, RJ – Brazil

**Keywords:** Graft Occlusion Vascular/complications, Thrombosis, Platelet Aggregation Inhibitors, Atherosclerposis/prevention and control, Risk Factors

Saphenous vein graft (SVG) stenosis or occlusion is the Achilles heel for a more liberal use of this type of graft in coronary artery bypass surgery (CABG) for myocardial revascularization.

During the first month after CABG, around 10% of SVG can be occluded due to surgical technique failure or thrombosis. At the end of the first year 15% of SVG can be occluded due to intimal hyperplasia. Perfect surgical technique, careful handling of the saphenous vein avoiding graft overdistension and aspirin use for life (or other antiplatelet agent) are important points for achieving a patent vein.^1^

After the first year, the most important etiology for SVG stenosis or occlusion is atherosclerosis, and after 10 years only about 60% of SVG are patent. Atherosclerotic SVG stenosis is the most common indication for CABG reoperation. The risk factors for SVG atherosclerosis and stenosis are similar to the ones in the native coronary artery.^1^

For SVG atherosclerosis prevention, the same strategy is used as that for native coronary artery atherosclerosis, namely risk factors modification and lipid-lowering medication.

The article “Impact of atherogenic indexes in saphenous vein graft stenosis”,^2^studied 534 patients through cineangiocoronariography performed at least one year after CABG (median 5.3 years). They divided the patients into two groups: 1- SVG(+), with at least one SVG with more than 50% stenosis with 259 patients; and 2- SVG(-), without any SVG stenosis with 275 patients. The authors studied lipid components of the plasma in both groups: total cholesterol (TC), triglycerides (TG), high-density lipoprotein cholesterol (HDL-C), non-high-density lipoprotein cholesterol (non-HDL-C) and low-density lipoprotein cholesterol (LDL-C). Based on those data they calculated the atherogenic index of plasma (AIP) and the atherogenic coefficient (AC). The AIP is calculated as the logarithm of the TG/HDL-C ratio. The AC is calculated by the simple formula (TC-HDL-C)/ HDL-C.

In the results section, they showed that diabetes mellitus, arterial hypertension, stroke, heart failure and the number of grafts were independent clinical parameters for SVG stenosis. Regarding the laboratory parameters, LDL-C, non HDL-C, HDL-C, AIP and AC were independent factors for SVG stenosis. Using paired comparisons of the ROC curve analysis, they have found no significant differences between AIP and AC, but both of them were better to predict SVG stenosis than HDL-C, LDL-C and non-HDL-C.

I have a few remarks and comments on this interesting paper, some of them already disclosed in the study limitations:

1. There was no clinical endpoint.
2. The use of lipid-lowering medication was low in both groups, being 59.8% in SVG(+) and 64.7% SVG(-). They found that the patients’ mean LDL-C levels were above the recommended ones for secondary prevention. Hata et al.^3^ have demonstrated that an aggressive treatment with statins to achieve an LDL<100 mg/dL decreases the number of affected grafts and the need for repeat revascularization.^3^
3. The number of grafts was an independent factor for SVG stenosis and the SVG (+)group had more implanted grafts than the SVG (-) group. As the authors consider that the main criterion to be included in the SVG(+) group was having at least one graft with more than 50% stenosis, I question whether studying patients with the same number of SVG would not be a more precise way of comparing the groups, as if a higher number of grafts are implanted, there is a higher probability of having at least one stenotic graft.
4. Regarding the no-touch technique for saphenous vein harvesting, the vein is removed from its bed with surrounding tissue, with a patency of 90% at 8.5 years being reported;^4^moreover, this type of vein harvesting was recommended in the 2018 European guideline for myocardial revascularization.^5^It would be interesting to know the behavior of AIP and AC in the medium-term results of those grafts.
